# Sedentary behaviour and adiposity in youth: a systematic review of reviews and analysis of causality

**DOI:** 10.1186/s12966-017-0497-8

**Published:** 2017-03-28

**Authors:** Stuart J.H. Biddle, Enrique García Bengoechea, Glen Wiesner

**Affiliations:** 1 Institute of Sport, Exercise & Active Living, Victoria University, Melbourne, Australia; 2 Institute for Resilient Regions, University of Southern Queensland, Education City, 37 Sinnathamby Boulevard, Springfield Central, QLD 4300 Australia; 3 McGill University, Montreal, Canada

**Keywords:** Sedentary, Screen time, Television, Children, Adolescents, Obesity, Weight status, BMI

## Abstract

**Background:**

Sedentary behaviour (sitting time) has becoming a very popular topic for research and translation since early studies on TV viewing in children in the 1980s. The most studied area for sedentary behaviour health outcomes has been adiposity in young people. However, the literature is replete with inconsistencies.

**Methods:**

We conducted a systematic review of systematic reviews and meta-analyses to provide a comprehensive analysis of evidence and state-of-the-art synthesis on whether sedentary behaviours are associated with adiposity in young people, and to what extent any association can be considered ‘causal’. Searches yielded 29 systematic reviews of over 450 separate papers. We analysed results by observational (cross-sectional and longitudinal) and intervention designs.

**Results:**

Small associations were reported for screen time and adiposity from cross-sectional evidence, but associations were less consistent from longitudinal studies. Studies using objective accelerometer measures of sedentary behaviour yielded null associations. Most studies assessed BMI/BMI-z. Interventions to reduce sedentary behaviour produced modest effects for weight status and adiposity. Accounting for effects from sedentary behaviour reduction alone is difficult as many interventions included additional changes in behaviour, such as physical activity and dietary intake. Analysis of causality guided by the classic Bradford Hill criteria concluded that there is no evidence for a causal association between sedentary behaviour and adiposity in youth, although a small dose-response association exists.

**Conclusions:**

Associations between sedentary behaviour and adiposity in children and adolescents are small to very small and there is little to no evidence that this association is causal. This remains a complex field with different exposure and outcome measures and research designs. But claims for ‘clear’ associations between sedentary behaviour and adiposity in youth, and certainly for causality, are premature or misguided.

## Background

Sedentary behaviour has been defined as low energy sitting (or reclining) during waking hours [1], thus excluding sleep or seated exercise. It is, essentially, ‘sitting time’ rather than ‘lack of exercise’. Research in this field has expanded exponentially since the early 2000s. Sedentary behaviour as a research topic has emerged on the basis of having shown high rates of sitting in contemporary society and associations with deleterious health outcomes [2]. Such associations have been claimed to be somewhat statistically independent of participation in moderate-to-vigorous physical activity (MVPA) [3, 4], and sedentary behaviour in adults has shown reasonable evidence of a causal relationship with all-cause mortality [5]. However, emerging evidence is also suggestive that the negative health effects of sedentary behaviour are more likely in those who are not sufficiently physically active [6].

Evidence on the health effects of sedentary behaviour started to build in a systematic way with studies from the 1980s on television (TV) viewing in children and adolescents. In 1985, Dietz and Gortmaker published a paper in which they suggested we may be ‘fattening our children at the TV set’ [7]. In analyses of data from over 6,500 young people from the National Health Examination Survey in the USA, they concluded not only that an association existed between TV viewing and adiposity, both cross-sectionally and longitudinally, but that their evidence “fulfils the criteria necessary to establish a causal association” (p. 811). Interestingly, they also state that TV viewing “only accounts for a small proportion of the variance of childhood obesity” (p. 811). Moreover, while claiming evidence for a dose-response relationship, their longitudinal data, shown in histograms, illustrates that more than five hours per day of TV viewing had the highest prevalence of obesity, whereas lower levels were less obviously indicative of a dose-response. This is suggestive of a stepped or threshold effect rather than a linear effect, the latter being shown a little more clearly for ‘superobesity’ (at or above 95^th^ percentile for triceps skinfold). The study by Dietz and Gortmaker did not control for dietary intake or physical activity.

Overall, Dietz and Gortmaker’s [7] study was an important start in recognising that one specific sedentary behaviour – TV viewing – might be a risk factor for obesity in young people. However, at the same time, some of the conclusions may have been premature on the assessment of causality.

Another key study was provided by Hancox et al. [8] in their investigation of over 1,000 children from New Zealand. They found that hours of weekday TV viewing between the ages of 5-15 years were associated with higher body mass index (BMI) some 10 years later. However, they also found similar associations for smoking, low fitness, and raised cholesterol. The data on smoking suggests that poor health behaviours may be clustering together rather than necessarily causing each other. Nevertheless, Hancox et al’s study provided more convincing evidence that TV viewing in childhood might be associated, prospectively, with adiposity later in life.

The first meta-analysis investigating associations between sedentary behaviour, in the form of TV viewing, and adiposity in youth was reported by Marshall et al. [9]. Reporting a fully corrected effect size (Pearson r) of 0.066, they concluded that while this was statistically significant, the very small amount of variance in adiposity explained by the amount of TV viewing “calls into question the clinical relevance” (p. 1241) of this association. Moreover, and especially when a wider range of sedentary behaviours is accounted for, opinions continue to be divided. For example, due to the use of cross-sectional designs and the potential confounding factors of physical activity and diet, Saelens [10] stated that the “conclusions regarding the relationship between sedentary behaviour and adiposity in youth are necessarily tentative” (p. 221). While Saelens was referring to a range of sedentary behaviours, most of the studies he reviewed concerned screen time, with nearly all including TV viewing. Nonetheless, several organisations have supported the view that TV viewing is a problematic behaviour from the stand point of obesity risk, including statements from the Australian College of Paediatrics [11] and an expert group of the American College of Sports Medicine [12].

Since the 1980s, but mainly from the mid-2000s, there has been expanding interest in whether sedentary behaviour is associated with negative health outcomes in both adults and young people. Given the developments in technology during this period, research has widened the focus from TV viewing to ‘screen time’ (TV and computers), and to other sedentary behaviours, including sitting at school, in a car, and pursuing other leisure interests. At the same time, technology has enabled researchers to assess the quantity and patterning of sedentary behaviour using wearable devices that assess either lack of movement or postural allocation. This has allowed for more diverse assessments alongside traditional self-reporting of time, behaviour, and context. Evidence seems to suggest that measures from wearable technology – so called ‘objective measures’ – yield much more inconclusive evidence for an association between sedentary behaviour and adiposity in youth, or even no association at all [13].

Key potential confounders of any relationship between sedentary behaviour and adiposity are physical activity and dietary intake, and with adolescents, maturational status. In addition, some have indicated that sleep may also play a role [14, 15]. Sedentary behaviours have sometimes been proposed to displace time in more active pursuits. Given that at any specific time, sitting precludes light, moderate or vigorous physical activity, this seems a logical assumption. However, the real issue is whether certain sedentary behaviours, or large amounts of sedentary behaviour, preclude physical activity at other times of the day. Given 24 h in a day, it is feasible that large amounts of both sitting and moving are possible. That is, they could co-exist over time. To date, typical findings suggest that less MVPA is associated with greater levels of sedentary behaviours, but this relationship is usually small. The association with light physical activity (e.g. standing and light ambulation) is large because this is where sitting time tends to get displaced to and from and the two behaviours are somewhat interdependent [16, 17].

Certain sedentary behaviours may also be associated with changed dietary patterns. For example, eating in front of the TV may trigger greater snacking or consumption of unhealthy foods prompted by advertisements. Pearson and Biddle [18] conducted a systematic review across all ages and concluded that greater sedentary behaviour, often studied as screen time, showed a clear association with unhealthy dietary intake, including higher consumption of energy-dense snacks and less consumption of fruits and vegetables. For children, “TV viewing was consistently inversely associated with fruit and vegetable consumption and positively associated with consumption of energy-dense snacks and drinks, total energy intake, and fast foods” (p. 185). Studies testing for associations between sedentary behaviour and adiposity, therefore, ideally need to control for levels of physical activity (i.e., MVPA), dietary intake, and possibly sleep.

A great deal of the literature has comprised observational and cross-sectional studies. To advance this field, we need to better understand to what extent sedentary behaviour might be causally associated with adiposity in youth. Claims of causality have been made (see earlier), but these have not been based on established criteria such as those proposed by Hill [19, 20]. Nine factors were listed by Hill (‘Bradford Hill criteria’): strength of association, consistency, specificity, temporality, biological gradient (dose-response), (biological) plausibility, coherence, experimental evidence, and analogy. For sedentary behaviour and adiposity in young people, the key issues that can be assessed using the findings from the current review of reviews are strength of association, consistency, specificity, temporality, coherence and biological plausibility, dose-response, and experimental evidence (see Table 3 in Results for definitions).

Given that sedentary behaviour is highly prevalent in modern society, that such behaviours (especially screen based) are rapidly evolving and changing, and obesity is a key health issue, it is important to try to resolve the inconsistencies evident in the literature on the association between sedentary behaviour and adiposity in young people. To this end, we conducted a review of reviews and an analysis of whether any such association can be judged as causal. The following research questions will be addressed:

1. Is there an association between sedentary behaviour and adiposity in young people?
2. If so, does this association differ by type of sedentary behaviour and type of marker of adiposity?
3. Do MVPA or dietary intake moderate this association?
4. To what extent can any association between sedentary behaviour and adiposity be considered causal when using key criteria proposed by Hill [19]?

Given the plethora of evidence available on the current topic, we conducted a review of reviews [21]. Such a method allows for a summary of evidence from multiple reviews that focus on the same but inevitably overlapping research questions, often using multiple methods and measures. In addition, it allows for a comparison of findings and to resolve discrepancies in conclusions that might exist across reviews. It also allows for an analysis of mediators and moderators of a relationship [21].

## Method

PubMed and Scopus were searched up to July 2016 to identify systematic reviews and meta-analyses examining relationships between sedentary behaviours and markers of weight status/adiposity in children and adolescents. Groups of thesaurus terms and free terms for sedentary behaviour (e.g. sedentary, sitting, TV viewing), markers of weight status (e.g. adiposity, BMI, obesity), age group (e.g. children, youth), and publication type (e.g. meta-analysis, synthesis) were used. This resulted in the following example search: title-abs-key(sedentary OR sitting OR “watching TV” OR “TV watching” OR “viewing TV” OR “TV viewing” OR “television watching” OR “watching television” OR “television viewing” OR “viewing television” OR “screen time” OR “computer use”) AND title-abs-key(weight OR obesity OR “body mass” OR BMI OR overweight OR adiposity OR fatness OR “body composition”) AND title-abs-key(children OR childhood OR youth OR adolescen* OR “young people”) AND title-abs-key(review OR meta-analysis OR meta-regression OR synthesis). Additional reviews and meta-analyses were identified by manually checking the reference lists of included papers and searching the authors’ own literature databases.

To be included in the present analysis, review papers had to meet the following criteria: 1) population to include children or adolescents under the age of 19 years; 2) include at least one measure of sedentary behaviour; 3) report associations of sedentary behaviours with a measure of weight status or adiposity; and, 4) be a systematic review or a meta-analysis. Reviews summarizing or quantifying the evidence for associations could be based on subjective (e.g. questionnaires) or ‘objective’ (wearable) measures of sedentary behaviour (e.g. accelerometers, inclinometers), as well as overall sedentary behaviour or setting-specific sedentary behaviour (e.g. TV viewing time). Reviews or meta-analyses including measures of sedentary behaviour that were a combination measure of sedentary behaviour and physical activity, such as categorical measures with sedentary as the least active category, were excluded. We also excluded pre-school children (usually less than 5 years of age) on the basis that their environmental and social context differs considerably from those attending school.

Only full text peer reviewed articles written in English were considered for inclusion. Titles and abstracts of the identified references were reviewed by two people to exclude articles out of scope. Subsequently, two reviewers independently reviewed the full text of all potentially relevant references for eligibility using a standardized ‘in-out’ form. Disagreements between these reviewers were discussed with a third reviewer and a consensus decision was reached.

Data extraction was conducted by three researchers; one checked all reviews and disagreements were settled by consensus. Where reviews covered multiple age groups, intervention types, behavioural measures or health outcomes, the extracted data were based on and limited to key inclusion criteria stated above.

The methodological quality of each systematic review was assessed using the ‘Assessment of Multiple Systematic Reviews’ (AMSTAR) rating scale [22]. AMSTAR contains 11-items to appraise the methodological aspects of reviews with items scored as “Yes”, “No”, “Can’t Answer” or “Not Applicable”. For AMSTAR items, see footnote to Table 2. The item on conflict of interest (COI) requires that the systematic review and all primary studies be assessed. We modified this item to assess only the review itself. PRISMA does not require a COI assessment for each primary study. Each of the included systematic reviews was assessed by one researcher, 30% by two researchers, and all assessments were discussed and agreed by three researchers.

Findings will be summarised as follows. First, results will be presented from reviews focussed on observational cross-sectional and longitudinal studies. Second, evidence from intervention studies will be presented. In both sets of results, data will be separated by self-report and objectively assessed sedentary behaviour. For self-reported behaviours, the type of behaviour will be specified. Where possible, outcome measures will be differentiated, such as reporting results for BMI or waist circumference. An appraisal of the causal nature of any relationships will then be assessed (e.g., strength of association). Emphasis will be given to systematic reviews that are more recent, larger, and have a higher AMSTAR rating.

One key aspect of interpretation of findings is regarding the strength of association. This is also dealt with in more detail later under our analysis of causality. In interpreting strength of associations or effects, we have drawn on multiple sources, including the interpretation by the original review paper authors, as well as the interpretation of others, such as Cohen [23] and Rosenthal [24].

## Results

### Characteristics of systematic reviews

**Fig. 1:**
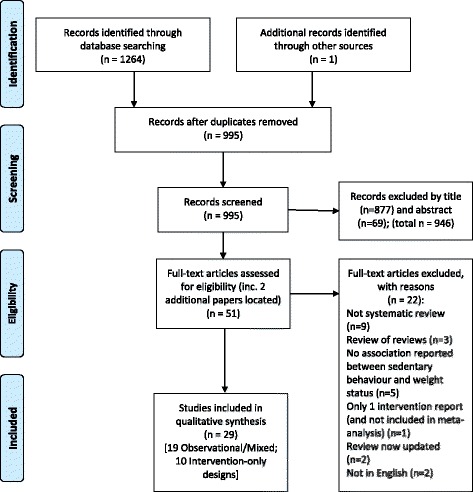
PRISMA flow diagram for selection of systematic reviews

Searches led to 51 full papers being assessed for eligibility, and 29 systematic reviews were selected for research synthesis (see the PRISMA flowchart in Fig. 1). Two of the main reasons for rejecting full papers at this stage were not being a systematic review and not having an association reported between sedentary behaviour and weight status.

Data from all reviews were extracted and summarized in Table 1. Nineteen reviews included data from primary studies utilizing observational methods [cross sectional (*n* = 4) [25–27], longitudinal (*n* = 5) [28–32], or both (*n* = 10) [9, 33–41]]. Four of these reviews also included data from intervention studies, with three only analyzing a single intervention [9, 40, 42], and one review including four. There were 10 reviews that focused on intervention studies [43–52]. Nearly all reviews included children and adolescents up to the age of 18 years, although not all age ranges were identical. Where possible, we extracted data and drew conclusions only from the appropriate age range (i.e. 6–18 years). The number of studies included in the systematic reviews varied greatly, ranging from 3 to 162 observational studies and 4 to 67 intervention studies. We estimated that the 29 reviews included 467 primary study papers, although due to poor reporting and errors in citations, this figure is not exact. One large review did not provide sufficient detail to list all primary studies used [49].

**Table 1:** Summary of the key characteristics of included systematic reviews

Author & date	Age range# (years)	Search dates	No. of studies reviewed on SB and WS (total and by design type)*	Sedentary behaviour(s) assessed	Weight status variable(s) assessed	Meta-analysis? (No. studies included^)	Quality assess-ment?	Conclusion reported	Comments
1. Reviews reporting on observational and mixed design methods									
Carson et al. (2016)[33]	5–17	From Feb 2010	162 [125 CS; 32 LG; 5 CC	TV, computer use, screen time, total SB	Various	No	Yes	Higher durations or frequencies of screen time and TV viewing were significantly associated with unfavourable measures of body composition across all study designs. But study quality rated very low to low. No associations between accelerometer assessed sedentary time, breaks and bouts and body composition.	Update of Tremblay et al. (2011) [61]
Cliff et al. (2016)[34]	2–18	To Nov. 2015	50 [37 CS; 9 LG; 4 CS + LG] (2–4 years = 3; 5–12 years = 37; 13–18 years = 10)	Objectively measured total SB, pattern of SB (i.e. breaks, bouts)	BF%, WC, BMI	Yes (19)	Yes	Overall: ‘no association’ for total volume of SB and adiposity. Longitudinal studies: level of evidence classification was ‘no association’. Cross-sectional meta-analysis: weak but significant positive association with high levels of heterogeneity observed.	Most studies measured total SB. For meta-analysis of adiposity, selection of coefficient followed hierarchy: 1) BF% 2) WC; 3) BMI.
Costigan et al. (2013)[35]	12–18	To Dec. 2011	19 [13 CS; 6 LG]	Screen-based SB: TV, video, computer, electronic gaming	BMI, body fatness, OW/OB	No	Yes	Strong evidence for a positive relationship between screen-based SB and weight status (especially for low risk-of-bias studies).	Leisure-time domain only. Girls only.
Fletcher et al. (2015)[36]	12–19	To Mar. 2014	21 (17 CS; 4 LG)	TV viewing, total screen time, computer use, and video game playing or video viewing (mostly self-report)	BMI (81% assessed objectively) Also: FMI and fat-free mass.	No	Yes	Moderate to strong evidence of the relationships between self-reported television viewing, total screen time and overall sedentary behavior with adiposity, independent of dietary intake.	Only included studies which adjusted for dietary intake.
Froberg & Raustorp (2014)[37]	6–19	Jan 2000 to Oct 2013	35 [28 CS; 7 LG]	Objectively assessed volume (total time) and patterns (bouts and breaks)	Various	No	Yes	Limited evidence for an association between objectively assessed volume of sedentary time with markers of obesity, when controlling for MVPA. Evidence on associations between sedentary bouts and breaks with weight status was inconclusive.	
Gorely et al. (2004)[38]	2–18	Not specified	24 [18 CS; 6 LG] (0-6y = 4; 7-18y = 20)	TV/video viewing time	Weight, body fatness	No	No	Body weight was positively associated with TV viewing time (4 samples) ‘Body fatness’ was unrelated to TV viewing time (association: 40% +; 61% no).	Excluded video/computer gaming.
Leech et al. (2014)[39]	5–18	To Nov 2012	6 [4 CS; 2 LG]	Sedentary behaviors (e.g. TV viewing, video watching, using the computer or internet and playing console games)	BMI	No	No	Findings to support an association between obesogenic cluster patterns (diet, PA, SB) and overweight and obesity were inconclusive with longitudinal research. Diet, PA and sedentary behavior cluster together in complex ways that are not well understood.	Despite the age group specified in the inclusion criteria, it is also noted that: “With the exception of one study [18] that included children aged 5–12 years, the ages of children and adolescents in these studies ranged from 9–21 years”.
Marshall et al. (2004)[9]	3–18	1985 - ?	30 Independent samples: TV: 52 [43 CS; 8 LG; 1 IN]. Computer games: 6.	TV viewing, video/computer game use	“Body fatness”: BMI, skinfold	Yes (30)	No	Small but statistically significant relationship between TV viewing and body fatness. Small and statistically non-significant relationship between video/computer game use and body fatness, but with small number of samples.	30 studies yielded 52 independent samples for the meta-analysis. Sample proportions by age: < 7 y (8%); 7–12 y (46%); 13–18 y (23%); combination (23%).
Mistry & Puthussery (2015)[25]	≤18	Jan 1990 to June 2013	5 (all CS) (All 5-18y)	TV and computer games	OW/OB	No	Yes	4/5 studies show positive correlation between TV/computer game time and weight status.	Selected studies all school-based. The review focussed on studies from SE Asia only, with the final included studies being conducted in India only.
Mitchell & Byun (2014)[40]	6–18	Jan 2008 - Sept 2012	63 [50 CS; 12 LG; 1 IN]	Self-reported SB (inc. screen time); objectively assessed SB	Various	No	No	Cross-sectional; screen time: 77% of studies show positive association with BMI; similar support for WC and fat mass. Cross-sectional; objective sedentary time: Small number of studies favour null association Longitudinal; screen time: favours association with changes in BMI and skinfolds Longitudinal; objective sedentary time: favours null association	Moderation analysis with 6 CS studies showed for all that screen time only associated with greater BMI if MVPA was low.
Must & Tybor (2005)[28]	<22	Not specified	15 (all LG)	Any measure of “Inactivity/SB”	Mostly BMI/BMI z-score. Also: skinfold, DEXA, BF% by BIA	No	No	Most studies (especially with younger subjects) showed a positive association of “inactivity/SB” with weight or adiposity outcomes.	Average follow-up ≥2 years. 9/15 studies focused on children <10 year.
Pate et al. (2013)[29]	5–18	Jan1990 - Jun 2012	4 (all LG)	Objectively measured SB (accelerometry)	Excessive fatness/body composition (adiposity, BMI, BMI-z, FMI, WC)	No	No	Mixed findings regarding association of SB with excessive fatness in children and adolescents. No association among younger children (5–9 years); positive association indicated between sedentary time and BMI in older children and adolescents (9–15 years).	Prospective cohort studies.
Prentice-Dunn & Prentice-Dunn (2012)[26]	2–19	2000–2010	9 (all CS) (11-18y;4-11y; 7-9y; Mean: 6.8y- SD:0.4;7-12y; 6y; 3-5y; 1-12y; Median:15y)	Parent-report or self-report of screen time, accelerometer counts, and direct observation	BMI and BF% (by various measures)	No	No	The majority of studies (7/9 studies) assessing sedentary behaviors (i.e. screen time) found a positive correlation with weight status. The 2 studies showing no association used objective measures of SB.	Inconsistent reporting of SB studies. Number based on Table 3 - summary of SB and WS findings.
Rey-Lopez et al. (2008) [41]	2–18	1990 - April 2007	78 [46 CS; 28 LG; 4 IN]	TV viewing, video games, computer use	Various	No	No	Cross-sectional: Positive association with OW/OB: TV viewing (k = 70 samples): 65–69% of studies Video games (k = 12): 50–67% Computer use (k = 18): 40–50% Longitudinal: Positive association with OW/OB: TV viewing (k = 45): 62–67% of studies Video games (k = 6): 67–100% Computer use (k = 4): 100% Interventions (k = 4): positive effects for weight status change in 1 of 4 studies.	
Saunders et al. (2016)[14]	5–17	To Jan 2015 (plus additional CINAHL search Jun. 2016)	10 (all CS) (6-18y = 9; mixed =1)	Any (accelerometer and screen-time)	BMI, WC, waist-to-height ratio, BF% (by BIA and DEXA), skinfolds.	No	Yes	A combination of high PA/low SB, compared with low PA/high SB, was associated with lower measures of adiposity and/or reduced risk of obesity.	Minimum sample size ≥300. SB considered in combination with PA and/or sleep.
Stierlin et al. (2015)[30, 42]	≤18	Jan. 2000 -May 2014	4 [3LG; 1 IN-RCT] [1 ‘toddlers & pre-schoolers’ (mean age 5y); 1 ‘children’ (mean age 6.3 and 10.3 for cohort 1 &2); 1 ‘adolescents’ (mean age 15.7y); 1 ‘children & adolescents’ (mean age ranging from 10.2–14.5y across countries).	Total SB time; subdomains of SB, inc. time spent watching TV, screen time, homework, reading. For studies using accelerometry, SB defined as <100 counts per minute.	Not reported	No	Yes	Screen time: positive association with weight status at follow-up (based on1 study). Total SB: no association with weight status (based on 3 studies).	Review of determinants of SB; excluded CS studies. “Consistent” evidence for weight status being positively associated with screen time comes from one study including two cohorts of young children (Fuller-Tyszkiewicz et al., 2012),
Tanaka et al. (2014)[31]	<19	1950s to Dec 2013	3 (All LG) Age range at baseline: 7-9y. Follow up period: 2–7 years.	Objectively measured SB	Various	No	Yes	No clear evidence that increased sedentary time is associated with increased adiposity.	
Van Ekris et al. (2016)[32]	≤18	To Jan 2015	50 (All LG)	TV, computer use, screen time, total SB	BMI, WC, BF, skinfolds, weight, weight for height, OW/OB)	Yes (8)	Yes	TV: strong evidence for positive association with overweight/obesity. Other outcome measures: insufficient evidence. Computer use/gaming: no or insufficient evidence. Screen time: strong evidence for BMI; moderate for OW/OB. Total SB: no evidence for BMI, WC, body fat. Insufficient evidence for OW/OB.	Update of Chinapaw et al. (2011). 50 studies on ‘anthropometrics’. Additional papers reviewed considered ‘multiple indicators of cardio-metabolic health’. These are not listed here.
Zhang et al. (2016)[27]	≤18	To June 2014	14 (all CS) (<6y = 3; 6-18y = 9; mixed = 2)	TV viewing time	“weight/height” (see Comments) For meta-analysis: OW/OB risk	Yes (14)	No (see Comments)	Increased TV watching is associated with increased risk of childhood obesity. A linear dose–response relationship was found for TV watching and childhood obesity, and the risk increased by 13% for each 1 h/day increment in TV watching.	Minimum sample size for inclusion >200. Unable to determine whether “weight/height” relates to specific assessment formula or just general categories. Publication bias assessed but not individual study quality.
2. Reviews reporting outcomes from interventions									
Azevedo et al. (2016)[43]	≤17	1980 – March 2015	67 [17 with 0-5 years.; 35 with 5-12 years.; 15 with 12-17 years.] (61 RCT or cluster RCT; 6 non-randomized CTs) [6 (SB only), 10 (SB + PA), 51 (SB + other behaviour(s))]	Activities undertaken whilst sitting or lying down, such as screen-based activities	Objectively measured BMI or BMI-z	Yes (51)	Yes	SB interventions were associated with a very small and clinically irrelevant effect on BMI or BMI-z when applied to the general population or normal weight population. By contrast, SB interventions to reduce BMI might be clinically effective for overweight/obese children.	Interventions targeted SB alone or combined with other behavioural components. Interventions appeared to be more successful when they were implemented with other behaviours (e.g. diet).
Bautista-Castano et al. (2004)[44]	≤18	Jan 1993 - Dec 2003	4 (all RCTs) (1 SB alone, 1 SB + PA, 2 SB + PA + diet) (11.7y, 5-7y, 8.9y, 8-10y)	‘Sedentary activities’, such as watching TV	BMI, triceps skin-fold, WC, WHR	No	No	Decreasing ‘sedentary activity’, such as watching TV, positively influenced the effectiveness of interventions designed to prevent childhood obesity	RCT studies with the school as the unit of randomisation, intervention and analysis.
De Mattia et al. (2007)[45]	Child-ren or adoles-cents (mean age 3.9 & 14.2 y)	1966 – Feb 2005	6 (all RCT’s) [1 (SB only), 2 (SB + PA), 1 (SB + diet), 2 (SB + PA + diet)] (Mean age: 10.4y; 10.0/10.2y; 14.2y; 3.9/4.0y; 8.9y; 9.5y/9.5y)	Recreational screen time	BMI, BMI-z, OW%, body composition (BIA, WHR, triceps skin-fold, DEXA scan)	No	Yes	SB interventions were associated with a modest improvement of weight parameters.	Controlled IN studies. As the SB messages in these interventions are often combined with other health information (e.g. healthy eating and exercise), it is not possible to estimate the magnitude of the weight influences because of SB messages alone.
Leung et al. (2012)[46]	6–19	1980 - Apr 2011	6 (1 SB only; 5 SB + Other)	Screen-based SB, “breaks from activity”, low EE activities (e.g. reading)	BMI, waist/hip circumference, BF%, skinfold thickness	No	No	Interventions targeting SB were effective at reducing SB and/or improving measurements related to weight status.	RCTs lasting ≥ 12 weeks; interventions aimed at reducing SB in school-aged children
Liao et al. (2014)[47]	≤18	To July 2012	25 (study design not specified) [(5 SB only), (10 SB + PA), (10 SB + PA + diet)] Mean age [5 (<6 years), 15 (6–12 years), 5 (>12 years)]	Watching TV/DVD/VCR, playing sedentary video/ computer games and general sitting time	BMI	Yes (25)	Yes	Interventions seeking to decrease sedentary behaviours among children significantly reduced BMI when compared with control groups; mean BMI mean difference (*g = −0.073,* *P* = 0.021) at post-intervention.	Multi-component interventions (SB + PA or SB + PA + diet) were not more effective in reducing BMI than SB interventions alone. Authors stated that although the mean BMI mean difference may not be considered clinically significant for the treatment of obese children, it may achieve public health significance in obesity prevention interventions among non-obese children.
Luckner et al. (2012)[48]	≤18	To Nov 2008	9 (7 RCTs; 2 controlled non-randomized) [(1 SB only- TV); (8 SB-TV + other, e.g., PA)]. No age breakdown provided in the meta-analysis.	TV viewing and other (not specified)	BMI or BF% (by skin-fold, BIA or DEXA)	Yes (8)	Yes	In children (0–18 years), the highest reductions in mean BMI were achieved through promoting reduced television viewing [-0.27 kg/m2 (95% CI -0.4 to -0.13 kg/m2)]. The meta-analysis suggested that interventions which aimed to reduce TV viewing led to a significant reduction in BMI.	Interventions targeting weight status. Studies conducted with general (i.e. non-obese) populations of children. Only 1 of the 9 studies aimed solely at reducing TV viewing; others also incorporated PA and other components.
Ramsey Buchanan et al (2016)[49]	All ages, inc. adults and ‘Child-ren’ mainly </=13y	1966 - June 2013	46 ‘behavioural interventions’ of’screen time’ and ‘screen time-plus’ studies (with children)	Recreational screen time	BMI, BMI-z, BF%	Yes (some calculations of effect sizes)	Yes	Reductions in BMI from screen-time-only interventions, mainly for ‘high intensity’ interventions. “Strong evidence that screen-time only interventions are effective at reducing recreational sedentary screen time … and improving or maintaining weight status”	IN’s primarily targeted recreational sedentary screen time. While review addressed all ages, most were with children.
Stice et al. (2006)[50]	≤22	1980 - Oct. 2005	5 (all with “random assignment”) [(2 SB + Ed), (3 SB + PA + Ed)] (mean age 8.9–11.7years)	Sedentary behaviours, such as media (TV, video games) use	Mostly BMI, skinfold thickness	Yes (5)	No	Sedentary behavior reduction (as a moderator) was not associated with significantly larger effects.	This meta-analysis focused solely on effect sizes for weight gain prevention effects. Inconsistency noted in the number of designated SB studies across various tables.
Wahi et al. (2011)[51]	≤18	To Apr. 2011	9 [2 (≤6 years); 9 (>6 years)] [6 SB-only; 1 SB + PA; 4 SB + PA + Diet]	Screen time (hrs/week)	BMI	Yes (6)	Yes	Interventions to reduce screen time were not effective and mean changes in BMI (−0.10 (95% CI: −0.28 to 0.09) not significant (*P* = .32).	RCTs aimed at reducing screen-time in children.
Wu (2016)[52]	≤18	To August 24, 2015	7 [<6 years = 2; 6–17 years = 5] [4 SB-only; 1 SB + PA; 2 SB + Diet]	Screen time (hours per week).	BMI	Yes (7)	Yes	Based on pooled analysis, including one with adults, interventions targeting screen time reduction had a significant effect on BMI reduction (-0.15 kg/m^2^, *P* <0.001) Notably, when looking specifically at 6–17 year olds, the mean BMI change was clearly not significant: -0.02 (95% CI: -0.18-0.15; P = 0.846).	Includes only RCT studies. According to authors, subgroup analyses stratified by baseline age and presence of co-interventions suggested no significant differences.

#Allowed as per review inclusion criteria. May not reflect characteristics of final included studies

*Number of studies specifically looking at both childhood/adolescent SB and WS within each review [which may differ from total number of included studies.]

^Number of studies (i.e. publications) represented in meta-analysis. Note that individual studies may have yielded multiple samples (e.g. males vs females) for the meta-analysis

*Abbreviations: BF* body fat, *BIA* bio-impedance analysis, *BMI* Body Mass Index (kg/m^2^), *BMI*-z BMI z-score (BMI standardised for sex and age), *CS* cross-sectional design, *CC* case control design, *DEXA* dual-energy X-ray absorptiometry, *EE* energy expenditure, *FMI* fat mass index, *IN* intervention(al) design, *LG* longitudinal design, *MVPA* moderate-to-vigorous physical activity, *OB* obese, *OW* overweight, *PA* physical activity, *RCT* randomised controlled trial, *SB* sedentary behaviour, *WC* waist circumference., *WHR* waist-hip ratio, *WS* weight status

Given that the field of research on sedentary behaviour and health outcomes is relatively new, the majority of reviews were published after 2011. Of the 19 focussing on observational studies, 15 (79%) were published between 2012 and 2016. The first reviews to include these topics were published in 2004, including a meta-analysis of associations between body fat and both TV viewing and computer use [9], and a review of correlates of TV viewing [38]. Of the reviews addressing the effectiveness of interventions, 7 of the 10 were published between 2011 and 2016. Overall, 4 of the observational reviews and 7 of the intervention reviews included a meta-analysis, or at least some calculation of effect sizes. Ten reviews included an assessment of study quality or risk of bias.

A key issue to address is the assessment of both exposure and outcome variables. For the measurement of sedentary behaviour, 17 reviews synthesised studies that had only self-reported behaviour, 4 for only objective measures [29, 31, 34, 37], and 8 reviews included both types of assessment. All reviews addressing self-reported sedentary behaviour included measures of screen time (TV viewing and computer time either singly or combined). Very few additional self-reported sedentary behaviours were assessed. For the use of objective wearable technology, all reviews relied on the Actigraph accelerometer, thus reporting on ‘low or lack of movement’ rather than sitting per se. A variety of outcome measures of weight status and adiposity were reported, with nearly all reviews reporting on BMI.

Table 2 summarises the AMSTAR ratings. From a total possible score of 11, the majority (66%) of reviews received a rating of at least 5. The ratings were partly a function of date of publication with 60% of those rated 4 or below and only 11% of those rated 5 or above being published prior to 2010. This is likely due to the more recent development and adoption of review guidelines (e.g. PRISMA).

**Table 2:** Methodological quality assessment of systematic reviews using the AMSTAR rating

	AMSTAR items											
Author (Year)	1	2	3	4	5	6	7	8	9	10	11*	Overall rating
Azevedo et al. (2016)[43]	Yes	Yes	No	No	No	Yes	Yes	Yes	Yes	Yes	Yes	8
Bautista-Castano et al. (2004)[44]	No	No	No	No	Yes	Yes	No	No	N/A	No	No	2
Carson et al. (2016)[33]	Yes	Yes	Yes	No	No	Yes	No	No	Yes	No	Yes	6
Cliff et al. (2016)[34]	Yes	Yes	Yes	No	No	Yes	Yes	Yes	Yes	Yes	Yes	9
Costigan et al. (2013)[35]	No	No	Yes	No	No	Yes	Yes	Yes	N/A	No	Yes	5
DeMattia et al. (2007)[45]	Yes	No	Yes	No	Yes	Yes	No	No	Yes	No	Yes	6
Fletcher et al. (2015)[36]	Yes	No	Yes	No	No	Yes	Yes	Yes	N/A	No	Yes	6
Froberg & Raustorp (2014)[37]	Yes	Yes	Yes	No	No	Yes	No	No	N/A	No	Yes	5
Gorely et al. (2004)[38]	No	No	Yes	No	No	Yes	No	No	N/A	No	No	2
Leech et al. (2014)[39]	No	No	Yes	No	No	Yes	No	No	N/A	No	Yes	3
Leung et al. (2012)[46]	No	No	Yes	No	No	Yes	No	No	N/A	No	Yes	3
Liao et al. (2014)[47]	No	No	Yes	No	No	Yes	Yes	Yes	Yes	Yes	Yes	7
Luckner et al. (2012)[48]	No	No	No	No	No	Yes	Yes	No	Yes	Yes	Yes	5
Marshall et al. (2004)[9]	No	No	Yes	No	No	No	No	No	Yes	No	Yes	3
Mistry & Puthussery (2015)[25]	Yes	No	Yes	No	No	Yes	Yes	Yes	Yes	No	Yes	7
Mitchell & Byun (2014)[40]	No	No	No	No	No	Yes	No	No	N/A	No	Yes	2
Must & Tybor (2005)[28]	No	No	C/A	No	No	Yes	No	No	N/A	No	No	0
Pate et al. (2013)[29]	No	Yes	No	No	No	Yes	No	No	N/A	No	Yes	3
Prentice-Dunn & Prentice-Dunn (2012)[26]	Yes	No	No	No	No	Yes	No	No	N/A	No	No	2
Ramsey Buchanan et al. (2016)[49]	C/A	Yes	Yes	Yes	No	Yes	Yes	Yes	Yes	No	Yes	8
Rey-Lopez et al. (2008)[41]	No	No	No	No	No	Yes	No	No	N/A	No	Yes	2
Saunders et al. (2016)[14]	No	Yes	Yes	No	No	Yes	Yes	Yes	C/A	No	Yes	6
Stice et al.(2006)[50]	No	No	Yes	Yes	No	Yes	No	No	Yes	No	Yes	5
Stierlin et al. (2015)[30]	Yes	No	Yes	No	No	Yes	Yes	Yes	N/A	No	Yes	6
Tanaka et al. (2014)[31]	No	No	C/A	No	Yes	Yes	Yes	Yes	N/A	No	Yes	5
Van Ekris et al. (2016)[32]	No	Yes	No	No	No	Yes	Yes	Yes	Yes	No	Yes	5
Wahi et al. (2011)[51]	No	Yes	Yes	Yes	No	Yes	Yes	Yes	Yes	No	Yes	8
Wu et al. (2016)[52]	No	Yes	Yes	No	No	Yes	Yes	No	Yes	Yes	No	6
Zhang et al. (2016)[27]	No	No	Yes	No	No	Yes	No	No	Yes	Yes	Yes	5

*Criterion modified to only assess conflict of interest/source of funding statement of the review

AMSTAR contains 11-items to appraise the methodological aspects of the systematic reviews. All 11-items were scored as “Yes”, “No”, “Can’t Answer” or “Not Applicable”. AMSTAR comprises the following items:

1. ‘a priori’ design provided;

2. duplicate study selection/data extraction;

3. comprehensive literature search;

4. status of publication as inclusion criteria (i.e., grey or unpublished literature);

5. list of studies included/excluded provided;

6. characteristics of included studies documented;

7. scientific quality assessed and documented;

8. appropriate formulation of conclusions (based on methodological rigor and scientific quality of the studies);

9. appropriate methods of combining studies (homogeneity test, effect model used and sensitivity analysis);

10. assessment of publication bias (graphic and/or statistical test); and

11. conflict of interest statement

### Evidence for an association between sedentary behaviour and adiposity from observational studies

For self-reported sedentary behaviour, all reviews addressed TV and screen time. All 7 reviews synthesising associations with TV viewing alone showed a positive relationship (i.e. greater TV viewing time associated with indicators of greater adiposity). The first review of correlates of TV viewing in youth showed that while greater TV viewing was associated with higher body weight, it was not associated with body fat [38]. Some of these associations are small, or very small, and this will be discussed in the analysis of causality.

Similar results were found for assessments of screen time, although all include TV viewing alongside video game/computer use. When analysing data from reviews that report results separately for computer use, however, the picture is less clear. Two reviews report no association [9, 32], while one review shows a clear association for longitudinal studies but mixed results for cross-sectional [41].

Results from longitudinal studies show some differences from those using cross-sectional designs. For example, van Ekris et al.’s [32] review of just prospective studies concluded that there was ‘strong’ evidence for an association with ‘overweight and obesity’ for TV viewing (‘strong’ was defined as two or more high quality studies showing consistent findings), but ‘insufficient’ evidence for TV viewing and other markers of adiposity. Similarly, they concluded ‘strong’ evidence for an association between screen time and BMI, but ‘insufficient’ for other measures of adiposity. However, a large number of cross-sectional studies reviewed by Carson et al. [33], concluded in favour of an association for adiposity with TV viewing and screen time.

All nine reviews assessing the association between objectively assessed ‘total’ sedentary time were consistent in their conclusion in showing a null or inconsistent association with adiposity. This was true for both cross-sectional and longitudinal designs. For example, Cliff et al.’s meta-analytic review [34] showed no association for longitudinal studies and a significant but very small association (*r* = 0.07), with high heterogeneity, for cross-sectional studies. Similarly, van Ekris et al. [32] concluded ‘no evidence’ of an association from prospective studies when using objective assessments of sedentary behaviour for measures of BMI, waist circumference, and body fat, with the majority of studies being rated as high quality.

Patterns of associations across different outcome measures were largely unclear when assessed across all study designs, although van Ekris et al’s [32] review of prospective studies showed ‘moderate’ or ‘strong’ evidence for an association for three out of four sedentary behaviour measures when the outcome was labelled as ‘overweight/obesity’. For BMI, the most commonly used outcome measure, results ranged from ‘no evidence’ (computer use/game time; objective sedentary time), ‘insufficient evidence’ (TV viewing; overall sedentary time), to ‘strong’ (screen time). Given that few studies adopted standard methods to assess actual body fatness or waist circumference and, instead, adopted self-reported or objectively assessed BMI, the role of the outcome measure is still unclear.

In summary, evidence from systematic reviews synthesising observational cross-sectional studies indicates an association between TV viewing, screen time and adiposity in youth, and some evidence, but less clear, for the use of computers. Results from longitudinal designs are less convincing and appear to be somewhat dependent on the nature of the exposure and outcome variables assessed. Regardless of design, there is no evidence for an association with adiposity for total sedentary time assessed using accelerometers.

The strength of associations will be analysed and discussed later in our analysis of causality. In addition, results concerning potential moderators and confounders will be considered after data are presented from reviews of intervention studies.

### Evidence for an association between sedentary behaviour and adiposity from intervention studies

Of the 10 systematic reviews reporting on adiposity effects from reductions in sedentary behaviour (all involving some form of screen time), 6 reported favourable changes in weight status [44–49] and 4 showed null or inconsistent effects [43, 50–52]. However, it is often difficult to assess the effects of interventions on adiposity outcomes from reductions in sedentary behaviour alone. Many interventions included additional behavioural components, such as physical activity and diet. For example, Ramsey-Buchanan et al’s [49] recent review of screen time and ‘screen time plus’ interventions showed that the majority included more than just screen time reductions. When considering 8 screen time-only intervention study arms, the BMI change ranged from -0.09 to -0.44, whereas from 37 screen time-plus intervention study arms it ranged from -0.08 to -0.21. While interventions considered more intense were seen to be more successful at behaviour change, the changes in adiposity and weight status could be considered quite modest. This is similar to results from an earlier review by DeMattia et al. [45] who concluded that interventions were “associated with improvement of weight parameters” but also stated that “the magnitude … is modest and is difficult to interpret, because normal BMI ranges vary with age and development in children” (p. 79). Wahi et al’s [51] review showed no effectiveness for interventions in reducing markers of adiposity, while the review by Wu et al. [52], when excluding one trial on adults, showed no change in BMI from six interventions.

A recent meta-analysis by Azevedo et al. [43] concluded that interventions are associated with changes in BMI/BMI-Z score, but these were reported as “very small” (SMD = -0.060, 95% CI: -0.098 to -0.022). The reduction in BMI was greater in those who were overweight, and interventions were more effective when implemented in children, as a multicomponent intervention, and delivered in a non-educational setting. Overall, interventions to reduce sedentary behaviour in young people have been shown to produce modest effects for weight status and adiposity.

### Analysis of evidence concerning causality

The degree to which sedentary behaviour and adiposity in young people can be assessed as being causally associated is mainly through the following ‘Bradford Hill criteria’ [19]: strength of association, consistency, specificity, temporality, coherence and biological plausibility, dose-response, and experimental evidence. Table 3 defines each of these factors and summarises key findings.

**Table 3:** Assessment of causality using assessments of strength of association, consistency, specificity, temporality, coherence and biological plausibility, dose-response, and experimental evidence

	Definition	Summary of evidence	
		Support?	
Strength of association	How strong is the association between sedentary behaviour and adiposity in young people?	Weak	Consistently low strength of association values from cross-sectional evidence for self-reported screen time and objectively assessed sedentary time (e.g., *r* < 0.01); values close to zero for BMI per additional hour/day of screen time in prospective studies; small significant and non-significant effects from interventions.
Consistency	How consistent is the evidence across different populations and in different settings?	Moderate-to-weak	Evidence on sex differences in inconclusive. Stronger evidence exists for an association in children than adolescents but this could be a function of the volume of research favouring younger age groups, as well as the issue of maturation confounding measures of adiposity. Evidence does not differ by country. Consistency across measures of sedentary behaviour and markers of adiposity is weak.
Specificity	Is adiposity mainly limited to the existence of sedentary behaviour?	No	It is clear that many factors can be listed that are associated with weight gain or higher levels of adiposity in young people. The factor of specificity, therefore, cannot be supported. However, Hill states that we must not overemphasise this issue because diseases may have more than one cause and that “one-to-one relationships are not frequent” (p. 297).
Temporality	Does sedentary behaviour precede the development of adiposity?	Weak	Reviews addressing prospective studies show a mixed pattern of results. Data on self-reported screen time have shown ‘strong’ evidence for an association with BMI, but ‘insufficient’ for other measures of adiposity. Evidence concerning objective measures of total sedentary behaviour is largely null.
Coherence and biological plausibility	Any interpretation of the data should not seriously conflict with what is known about weight status and adiposity in young people. Biological plausibility provides further support for causation.	Moderate	While it is plausible and coherent with current knowledge that low energy expenditure in the form of sitting could be obesogenic, often these behaviours (e.g., TV viewing) co-exist with other behaviours. These might include excessive dietary intake and prompts from TV advertising for unhealthy foods. Individual sedentary behaviours are usually correlated in only a small way with moderate-to-vigorous physical activity, thus it cannot be claimed that one sedentary behaviour automatically precludes being physically active over time.
Dose-response	Do higher levels of sedentary behaviour show higher levels of adiposity?	Yes	Two reviewers provide support for a dose-response relationship. Carson et al. showed that more or less regardless of how TV viewing categories were compared, higher viewing was associated with greater adiposity. Zhang et al. calculated an odds ratio per 1 h/day increment in TV watching as 1.13 (95% CI 1.03–1.19). Graphical data suggested a linear relationship.
Experimental evidence	Is there evidence from interventions using experimental methods for changes in adiposity to result from changes in sedentary behaviour?	Weak	The analysis we have undertaken in this review of reviews summarises the evidence concerning effectiveness from interventions as ‘modest’, although some groups (e.g., obese) may gain more benefit. Effect sizes from meta-analyses, however expressed, are mostly small and both significant and non-significant.

Support for *strength of association* is weak. Starting from the first meta-analysis investigating TV viewing and body fatness in youth by Marshall et al. [9] (*r* = 0.066), to a recent review concerning objective measures of sedentary behaviour by Cliff et al. [34] (*r* = 0.07), effect sizes are very small, though both are significant. When comparing highest versus lowest TV categories, Zhang et al. [27] calculated an odds ratio of 1.47 for obesity, considered just below the lower threshold for ‘moderate’ strength [24]. However, this value is similar to that for sedentary behaviour and all-cause mortality in adults [5]. In a statistical integration of nine prospective samples, van Ekris et al. [32] reported an effect close to zero for the relationship between baseline TV viewing and BMI at follow-up when controlling for baseline BMI. Inspection of their Forest plot shows large variation, with 3 of the samples showing significant effects, but overall a range of beta values from -0.02 to 0.317. Some reviews only report the direction rather than strength of association [35].

The strength of effect from interventions is small-to-moderate, with some effects significant and others not. The largest effects are for high intensity screen time interventions on BMI (median reduction = -0.44) [49]. The most recent meta-analysis of 51 interventions [43] has reported an effect size (SMD) of only -0.06, although this was significant. This is similar to Liao et al’s [47] result (Hedges’ g = -0.073) which was also significant. Overall, therefore, strength of association is small, although sometimes significant. This raises the issue of whether such values reflect clinically meaningful or practical values.

Evidence for *consistency* of findings is moderate-to-weak. Consistency across demographics, such as age and sex, is reasonable, while it is less consistent for different sedentary behaviours and markers of adiposity. While the evidence is somewhat supportive of stronger associations for younger children, it is not clear if this is due to the confounding of maturational status in older cohorts.

Evidence for *specificity* is clearly not supported. One cannot find, nor would one expect, data supporting the view that obesity is primarily due to sedentary behaviours rather than a lack of MVPA or unhealthy dietary intake. Hill [19] states that this does not necessary preclude a conclusion supporting causation as it is rare to find a disease or condition (i.e. obesity) to have a single behavioural cause.

Support for the *temporal sequence* from sedentary behaviour leading to adiposity outcomes is weak. Using prospective data for both self-reported screen time and objectively assessed total sedentary time, the evidence appears to be mixed. For example, screen time has been shown to have ‘strong’ associations with BMI, but ‘insufficient’ evidence exists for other adiposity measures. Moreover, objective measures preceding assessments of adiposity show largely null findings. It is plausible, though rarely tested in young people, for higher levels of adiposity to lead to greater sedentary behaviour – so-called ‘reverse causality’. Indeed, it is plausible also for ‘reciprocal causality’ with a cycle of higher obesity, higher sedentary behaviour, and further increases in adiposity, thus making it difficult to know what might come first. In addition, with a few exceptions, follow-up periods in prospective studies are often quite short compared to those for adults and all-cause mortality [5]. From the 50 prospective studies reviewed by van Ekris et al. [32], follow-up averaged 3.15 years (range: 8 weeks to 8 years), with 84% less than 5 years, and nearly half less than 2 years.

There is moderate support for *coherence and biological plausibility* in the research on young people. Logically, low energy expenditure from periods of sitting should be associated with measures of adiposity, overweight and obesity. However, many studies do not adequately control for important potential confounders, such as physical activity, diet or maturational status. With evidence showing only small associations between MVPA and sedentary behaviour [17], it is proposed that both can co-exist across the day. This means that we need to control for MVPA or analyse data for those differing in levels of MVPA. It is the former strategy that has mainly been adopted, if indeed physical activity is controlled for at all. However, given that the associations between sedentary behaviour and adiposity are small, attenuation effects for MVPA may be limited. In Mitchell and Byun’s review [40], all 6 cross-sectional studies where a moderation analysis was conducted showed that associations between screen time and BMI were only evident when MVPA was low. In addition, research suggests that the difference in energy expenditure between sitting and standing still is very small [53].

Froberg and Raustorp’s [37] review of objectively assessed sedentary behaviour showed that adiposity was largely unrelated to sedentary behaviour even when controlling for levels of MVPA. Costigan et al’s [35] review of adolescent girls showed in a summary table that nine of 10 cross-sectional studies reported a positive association between screen time and weight status, but only five controlled for physical activity (and three studies appeared to be missing from the analysis). For longitudinal studies, they concluded all six had positive associations, with only half controlling for physical activity. From their table of results, only one longitudinal and two cross-sectional studies reported positive associations between screen time and weight status, controlled for physical activity, and had a low risk of bias rating. But the overall conclusion from this review was that a ‘strong’ association existed.

Unhealthy dietary intake has been shown to be associated with higher levels of screen time (including TV viewing) for adults, adolescents and children [18]. Some have argued that this may be the mechanism accounting for any association between screen time and adiposity. Few studies have controlled for dietary intake in their assessment of the association between sedentary behaviour and weight status. Contrary to expectations stemming from the reviews on screen time and dietary intake, Fletcher et al. [36] showed that any associations between screen time and adiposity were largely independent of diet. However, the variability in assessments of different dietary items was large. Moreover, the strength of association between key variables was not reported.

Studies in which statistical clustering of diet, physical activity and sedentary behaviour were reviewed by Leech et al. [39] but they found none that looked only at the clustering of sedentary behaviour and diet. When all three behaviours were assessed, clusters were shown to be associated with adiposity in adolescents. The independent or synergistic roles of sedentary behaviour were not clear.

In conclusion, there is biological plausibility as well as some level of coherence in showing TV viewing to be associated with a less healthy diet and greater snacking. However, the evidence is only moderate due to a lack of data testing whether true moderation effects exist for those who are physically active or inactive, and those who consume largely healthy or unhealthy diets. It is equally plausible that levels of adiposity will only be associated with sedentary behaviour for those who are inactive and have unhealthy diets. Sleep patterns may also need to be accounted for [15].

*Dose-response*, or biological gradient, is an important factor for determining causality. While dose-response curves can take on different shapes, and some associations may be more of a stepped or threshold function than a linear one, some gradient might be expected. From two analyses of dose-response, there is support for a gradient. For example, Zhang et al’s [27] review is the only meta-analysis to directly test for a dose-response effect, and they show that adiposity increases for each additional hour of TV viewing by odds of 1.13. Their graphical interpretation of the data suggests a linear effect. This is somewhat contradicted by the data from van Ekris et al [32] who report close to zero effects on adiposity for each additional daily hour of screen time. Carson et al. [33] showed that higher categories of TV viewing were associated with higher levels of adiposity. Given the analysis by Zhang et al., we conclude that there is evidence for a dose-response association between sedentary behaviour and adiposity in youth, however the magnitude of this trend appears to be small.

Finally, we conclude that there is only weak evidence from *experimental designs* for an effect of sedentary behaviour on changes in adiposity. Most effects are modest at best, with some being null. Some populations, such as those already obese, may have more to gain and show greater experimental effectiveness.

In summarising our assessment of the evidence concerning the association between sedentary behaviour and weight status or adiposity in young people, we conclude that there is no evidence to support causality. As shown in Table 3, evidence for strength of association is weak, consistency is moderate-to-weak, specificity is not supported, temporality is weak, coherence and biological plausibility is moderate, experimental evidence is weak, but there is evidence for a small dose-response association.

## Discussion

The purpose of this synthesis was to critically analyse the voluminous literature concerning the association between sedentary behaviour, usually in the form of either screen time or objectively assessed total sedentary time, and markers of weight status or adiposity in children and adolescents. Our research synthesis was conducted to answer four research questions. First, is there an association between sedentary behaviour and adiposity in young people? Our conclusion is that this association has been demonstrated but is small and somewhat inconsistent. Second, does this association differ by type of sedentary behaviour and type of marker of adiposity? Our conclusion is that screen time behaviours show some associations, but not objectively assessed total sedentary time. BMI has been studied most often but it is difficult to make clear comparisons between outcome measures.

Third, do MVPA or dietary intake moderate this association? Our conclusion is that too few studies control for dietary intake. One review of 21 observational studies concludes that sedentary behaviour is associated with adiposity in youth independently of dietary intake while another review has shown that screen time is associated with consumption of a less healthy diet. Studies controlling for MVPA have shown mixed findings, with some evidence of attenuation, but very few studies have tested for moderation effects. Those that have supported effects for adiposity from sedentary behaviour are mainly for those young people low in MVPA [40].

Finally, to what extent can any association between sedentary behaviour and adiposity be considered causal? Our conclusion is that while there is some evidence for a small dose-response relationship, other key factors, including strength of association and experimental evidence, is largely weak. Causality cannot be established given the current evidence base.

This popular and quite long established field of research is replete with uncertainty. Dietz and Gortmaker [7] claimed that TV was causally associated with body fatness in young people, yet this was based on only one, albeit large, observational study. Subsequently, the meta-analysis by Marshall et al. [9] showed a significant but very small effect size for mainly cross-sectional studies, claiming that it was likely to be of limited clinical relevance. But a recent very large international cross-sectional study of over 207,000 adolescents and 77,000 children from 54 countries concluded that TV viewing was associated with BMI in a dose-response fashion [54]. The authors of this study also stated that their conclusion supported two of the reviews we have assessed in the present omnibus review [9, 41]. However, the review by Marshall et al. [9], as just stated, showed a very small association, while the review by Rey-Lopez et al. [41] did not quantify the strength of association nor test for dose-response effects. In short, there appear to be numerous interpretations of the nature and importance of any association between sedentary behaviour and adiposity in young people.

### Are associations ‘sufficient’ and meaningful?

It is clear that many studies do report positive associations between sedentary behaviour and markers of adiposity in young people when the behaviour is self-reported as some form of screen time. But, as we discussed in the assessment of strength of association, the magnitude of this relationship is small. This was not disputed by Dietz and Gortmaker [7]. Marshall et al. [9] questioned whether such an association was clinically relevant, while Hancox and Poulton [55], in an analysis of longitudinal data on TV viewing and BMI, concluded that while the effect is small, there is a case to be made in support of the importance of TV viewing for childhood obesity.

There seems to be no dispute that the strength of association for screen time is small. The key issue is how meaningful is this association? Given that nearly all children and adolescents watch TV and use computers, a small ‘effect’ across a very large population could be significant for public health. On the other hand, analyses are mainly with those in healthy ranges of BMI. Moreover, children and adolescents are usually considered the most active and ‘healthy’ segment of society and, notwithstanding current obesity data, are largely free of non-communicable diseases common in adults. This raises the issue of whether we should expect much of an association.

In Liao et al.’s meta-analysis [47] of sedentary behaviour interventions, they concluded “Although the observed magnitude of BMI mean differences (*g* = −0.073, *P* = 0.021) between intervention and control groups at post-intervention may not achieve a level considered to be clinically significant (a minimum of 0.25 standardized BMI unit reduction) for the treatment of obese children, *it may be approaching the magnitude of change required to achieve population-level public health significance in obesity prevention interventions among non-obese children*, which is not entirely known” (p. 164; emphasis added).

Hancox and Poulton [55] raise an important point in suggesting that the small associations for screen time and adiposity in youth may not be too different from other associations that we seem to accept as meaningful. They cite three studies supporting their view that physical activity is related to BMI in only a small way, or not at all. That said, Ness et al. [56], while reporting data from both objective physical activity and fat mass (using a dual x-ray emission absorptiometry - DEXA), concluded that there was a “strong negative dose-response association” (p. 481) with the most adjusted model accounting for TV viewing as a confounding variable.

As stated in our results, Zhang et al. [27] reported an odds ratio of 1.47 for obesity from cross-sectional TV viewing studies, when comparing highest versus lowest TV categories. While this is just below the lower threshold for ‘moderate’ strength [24], it does suggest an association between more extreme groups. On the other hand, van Ekris et al. [32] reported an effect close to zero for TV viewing and BMI prospective studies and only 3 of 9 samples showed significant effects.

Even with small effects, screen time may still be important for adiposity over time. As we have reported, Hancox et al. [8] showed prospective associations of TV viewing on adiposity over the transition into young adulthood, and Thorp et al. [57], in reviewing adult studies, concluded that there was a consistent relationship between sedentary behaviour and weight gain from childhood into adulthood. These findings suggest that we need to know more about the tracking of sedentary behaviour into adulthood [58] and the possible cumulative effect of such behaviours on adiposity.

In summary, the issue of strength of association and the ‘meaningfulness’ of any association remains a contentious issue. There is agreement that associations and effects are essentially small, yet the interpretation of these, and their public health importance, is still open to debate.

### Can we rule out alternative explanations?

Given the extensive number of cross-sectional studies, some of which have been large and influential [7, 54], and the mixed conclusions from prospective studies [32], it is plausible that young people with greater adiposity choose to engage in higher levels of sedentary behaviour. This ‘reverse causality’ hypothesis has rarely been properly tested, but remains plausible, as does a ‘reciprocal causality’ cycle. That said, there are studies showing some direction of effect from longitudinal studies [8], increasing confidence that it could be screen time preceding adiposity. But overall, our conclusion concerning the temporal sequencing of effects remains weak.

Another plausible explanation is that the association between sedentary behaviour and adiposity is a spurious one, caused by other co-existing variables. The main candidates are physical activity and dietary intake, and possibly sleep. For moderate-to-vigorous physical activity, the evidence is mixed, with some studies showing associations between sedentary behaviour and adiposity even when controlling for MVPA, but others suggesting the effects are only evident for those with low MVPA. The latter would suggest that MVPA can over-ride the negative effects of sedentary behaviour, but with many people not meeting recommended levels of MVPA, sedentary behaviour may still be important.

The evidence concerning diet is also somewhat mixed. It has been shown that screen time is associated with less healthful eating [18], yet one review concludes that associations between sedentary behaviour and adiposity are not affected by diet [36]. Limitations of the studies in this field include the measures of diet, and timing of assessments. Dietary intake is notoriously difficult to assess through self-report and measurement error will be high. Moreover, studies have not assessed diet contemporaneously with specific sedentary behaviours. All we can conclude from studies is that some sedentary behaviours (e.g. TV viewing) are statistically associated with markers of dietary intake. We are not able to conclude that the sedentary behaviour occurred at the same time as unhealthy eating or that the two are causally linked. It is equally plausible that unhealthy snacking, for example, is part of a wider set of clustered unhealthy behaviours, such as high sedentary behaviour, low MVPA, poor sleep hygiene, and smoking.

In conclusion regarding alternative explanations, there is some evidence that sedentary behaviour is not harmful to health for those who are sufficiently physically active, showing that any associations with adiposity cannot be seen as totally independent of MVPA, and that dietary patterns may also co-exist with sedentary behaviour. In both cases the evidence is somewhat inconclusive, suggesting that we cannot yet rule out alternative explanations.

### What might be explaining associations between sedentary behaviour and adiposity?

Although the association between sedentary behaviour and adiposity is, in our analysis, quite weak, there is evidence that some association does exist for screen time. Given that conclusion, we need to identify potential mechanisms for such effects. The most obvious is that sedentary behaviours are, by definition, low energy expenditure behaviours [1]. But the key issue is energy expenditure across the day and the levels of energy intake. This is why levels of physical activity must be accounted for alongside diet.

There has often been an assumption that sedentary behaviour displaces more physically active pursuits, and this explains some of the links to obesity. However, while engaging in a sedentary behaviour inevitably precludes physical activity at that specific time, we know that levels of MVPA are only related to sedentary behaviours in a small way. This refutes the so-called ‘displacement hypothesis’. Such a hypothesis would be supported only if we can demonstrate that people engaging in high levels of sedentary behaviours are also less physically active. This has not generally been shown. Over a 24-h period we can take part in a variety of behaviours, ranging from sleep, sedentary, light activity, and MVPA, to highly vigorous physical activity. Being sedentary will most likely displace some light activity (e.g. sitting and standing are mutually exclusive and somewhat co-dependent), but reducing the time sitting in a day may have little effect on how much MVPA is undertaken. Future research must take into account the co-dependence of constructs across a movement continuum which includes sleep, sedentary behaviour, light physical activity, and MVPA. At any one time, an individual can only do one of these behaviours. Changes in one (e.g. TV viewing) must be displaced to another [59].

With the evidence that screen time (including TV viewing) is more likely to show an association with adiposity than overall sedentary time, this suggests that messages emanating from screens, such as advertisements for unhealthy foods, might be a factor. This could be coupled with more ‘mindless’ eating in front of the TV and therefore possible over-consumption. Strasburger et al. [60] concluded that the media have a significant impact on adolescent’s eating behaviours, and that parents should be encouraged to turn off the TV during mealtimes. But equally, they say that the role of media is complex and is in need of further research.

Measurement issues are important in understanding our findings, including both the measurement of exposure and outcome variables. With our clear conclusion that objectively assessed sedentary time is largely unrelated to markers of adiposity in youth, we can conclude that either sedentary behaviour throughout the day is not important for adiposity, or that only certain types and contexts of sedentary behaviour are influential. The latter infers that it is not just sitting that is a risk factor for greater adiposity, but the type of sitting and its coupling with other behaviours. But it might also suggest a measurement issue. For example, if screen viewing shows slightly higher and more consistent associations with weight status, this could be because it is easier to recall the amount (and possibly context) of this behaviour. Most people know the length of TV programs, for example, but may struggle to recall time spent reading or driving in a car.

BMI is the most common measure of weight status used in the various reviews. Whilst simple and useful at a population level for the estimation of overweight/obesity levels in adults, its use in children is confounded by the growth and maturation issues associated with significant anthropometric changes in a relatively short period. To address this, relative BMI z-scores (standard deviation scores) have been developed to account for a child’s age and sex. Several of the reviews we analysed included BMI-z as a measure of obesity, however few reviews specifically analysed this. Moreover, the issue of pubertal maturation is particularly troublesome when considering longitudinal studies in children and adolescents. Significant changes in relative proportions of lean and fat mass may confound results if not adjusted for in a meaningful way. This may be one reason why longitudinal evidence in younger, pre-pubertal children may be a little stronger. Must and Tybor [28], for instance, concluded that most studies - especially with younger participants - showed a positive association of “inactivity/sedentary behaviour” with weight or adiposity outcomes. By contrast, Pate et al [29] concluded that there was no association among younger children (5-9 years) but a positive association between sedentary time and BMI in older children and adolescents (9-15 years). Stierlin et al [30] concluded that “consistent” evidence existed for weight status being positively associated with screen time but the evidence stemmed from one study including two cohorts of young children. In future, studies will need to account for maturational status to aid interpretation of weight status results in young people.

Finally, Hancox and Poulton [55] raise an interesting issue that while the association between TV viewing and adiposity might be small, this could be an under-estimation due the lack of young people who watch no TV at all. In other words, all analyses involve young people who watch at least ‘some’ TV and either compare them with those who watch a great deal more, or calculate associations using TV viewing time that is represented by a truncated range of values (i.e. does not include values at or near zero).

## Conclusions

Using the evolving methodology of a systematic ‘review of reviews’, we were able to provide the following answers to our main research questions:

1. *Is there an association between sedentary behaviour and adiposity in young people? If so, does this association differ by type of sedentary behaviour and type of marker of adiposity?* Evidence from observational cross-sectional studies indicates a small association between TV viewing, screen time and adiposity in youth, and some evidence, but less clear, for the use of computers. Results from longitudinal designs are less convincing and appear to be somewhat dependent on the nature of the exposure and outcome variables assessed. There is no evidence for an association with adiposity for total sedentary time assessed using accelerometers. Interventions have been shown to produce modest effects for weight status and adiposity. Effects may be greater in more obese populations. Most studies assessed BMI, but no clear conclusions can be drawn concerning diverse results resulting from the assessment of different outcome measures.
2. *Do MVPA or dietary intake moderate this association?* TV viewing has been shown to be associated with a less healthy diet and greater snacking although evidence for true moderation is still unclear. Evidence is emerging suggesting that greater adiposity (and other negative health effects) may be most pronounced for those not engaging in high amounts of MVPA.
3. *To what extent can any association between sedentary behaviour and adiposity be considered causal when using key criteria proposed by Hill* [19]*?* There is no evidence to support a causal association between sedentary behaviour and weight status in young people. Evidence for strength of association is weak, consistency is moderate-to-weak, specificity is not supported, temporality is weak, coherence and biological plausibility is moderate, experimental evidence is weak, but there is evidence for a small dose-response association.

This remains a complex field with different exposure and outcome measures and research designs. But claims for ‘clear’ associations between sedentary behaviour and adiposity in youth, and certainly for causality, are either premature or misguided.
